# Glial metabotropic glutamate receptor-4 increases maturation and survival of oligodendrocytes

**DOI:** 10.3389/fncel.2014.00462

**Published:** 2015-01-14

**Authors:** Simona Federica Spampinato, Sara Merlo, Mariangela Chisari, Ferdinando Nicoletti, Maria Angela Sortino

**Affiliations:** ^1^Department of Biomedical and Biotechnological Sciences, Section of Pharmacology, University of CataniaCatania, Italy; ^2^Department of Physiology and Pharmacology, University of Rome SapienzaRome, Italy; ^3^IRCSS NeuromedPozzilli, Italy

**Keywords:** multiple sclerosis, experimental autoimmune encephalomyelitis, astrocytes, transforming growth factor β, oligodendrocytes

## Abstract

Group III metabotropic glutamate (mGlu) receptors mediate important neuroprotective and anti-inflammatory effects. Stimulation of mGlu4 receptor reduces neuroinflammation in a mouse model of experimental autoimmune encephalomyelitis (EAE) whereas mGlu4 knockout mice display exacerbated EAE clinical scores. We now show that mGlu4 receptors are expressed in oligodendrocytes, astrocytes and microglia in culture. Oligodendrocytes express mGlu4 receptors only at early stages of maturation (O4 positive), but not when more differentiated (myelin basic protein, MBP positive). Treatment of immature oligodendrocytes with the mGlu4 receptor agonist L-2-Amino-4-phosphonobutyrate (L-AP4; 50 μM for 48 h) accelerates differentiation with enhanced branching and earlier appearance of MBP staining. Oligodendrocyte death induced by exposure to 1 mM kainic acid for 24 h is significantly reduced by a 30-min pretreatment with L-AP4 (50 μM), an effect observed only in the presence of astrocytes, mimicked by the specific mGlu4 receptor positive allosteric modulator N-Phenyl-7-(hydroxyimino)cyclopropa[b]chromen-1a-carboxamide (PHCCC) (30 μM) and prevented by pretreatment with the mGlu4 receptor antagonist, cyclopropyl-4-phosphonophenylglycine (CPPG) (100 μM). In astrocytes, mGlu4 receptor is the most expressed among group III mGlu receptors, as by Quantitative real time PCR (QRT-PCR), and its silencing prevents protective effects. Protection is also observed when conditioned medium (CM) from L-AP4-pretreated astrocytes is transferred to oligodendrocytes challenged with kainic acid. Transforming growth factor β (TGF-β) mediates the increased oligodendrocyte survival as the effect of L-AP4 is mimicked by addition of 10 ng/ml TGF-β and prevented by incubation with a neutralizing anti-TGF-β antibody. In contrast, despite the expression of mGlu4 receptor in resting and activated microglia, CM from L-AP4-stimulated microglia does not modify kainate-induced oligodendrocyte toxicity. Our results suggest that mGlu4 receptors expressed in astrocytes mediate enhanced survival of oligodendrocytes under conditions of excitotoxicity.

## Introduction

Oligodendrocyte damage is a critical step in neuroinflammation occurring in demyelinating diseases such as multiple sclerosis (MS; Lassmann, 1998). Oligodendrocytes express several glutamate receptors including ionotropic glutamate receptors that mediate excitotoxicity, but also metabotropic glutamate (mGlu) receptors, G-protein-coupled receptors that are classified in three different families depending on their pharmacological profile and signaling pathway (Nicoletti et al., 2011). These include group I (mGlu1 and mGlu5) receptors coupled to phosphoinositide hydrolysis, group II (mGlu2 and mGlu3) and group III (mGlu4, mGlu6, mGlu7, mGlu8) receptors, all negatively coupled to adenylate cyclase (Conn and Pin, 1997).

Several mGlu receptors seem to play a role in neuroinflammation and changes in their expression have been reported in MS brain. Specifically, mGlu1 receptor shows an enhanced axonal immunoreactivity (Geurts et al., 2003) and appears in reactive microglia and astrocytes in MS white matter (Newcombe et al., 2008). Similarly, mGlu2/3 receptors are markedly overexpressed in astrocytes in acute and chronic active MS lesions (Geurts et al., 2003), staining large reactive astrocytes as well as small round cells, very likely glial progenitors (Newcombe et al., 2008). More controversies exist regarding the expression of mGlu5 receptors that, however, are described to be enhanced in reactive astrocytes of MS lesions (Geurts et al., 2003). Finally, mGlu8 receptor immunoreactivity is increased predominantly in cells of the microglia/macrophage lineage in actively demyelinating lesions and occasionally in reactive astrocytes (Geurts et al., 2005), whereas mGlu4 receptor is overexpressed in a selected population of reactive astrocytes localized in the rim of chronic active lesions in MS brain (Geurts et al., 2005).

Much attention has been focused on the involvement of group III mGlu receptors in neuroinflammation since these receptors are expressed in glial cells *in vitro* and their activation inhibits the production of inflammatory chemokines (Besong et al., 2002), the release of glutamate and superoxide (McMullan et al., 2012; Mead et al., 2012) and reduces astroglial and microglial neurotoxicity (Taylor et al., 2003; Zhou et al., 2006; Pinteaux-Jones et al., 2008). In addition, activation of group III mGlu receptors reduces the disability score in mice with experimental autoimmune encephalomyelitis (EAE), an established animal model of neuroinflammation and demyelination (Besong et al., 2002). More importantly, mGlu4 receptors are expressed in peripheral dendritic cells and mediate adaptive immunity (Fallarino et al., 2010). mGlu4 receptor knockout mice are more vulnerable to EAE and treatment of wild type animals with a mGlu4 receptor enhancer reduces EAE by modulating the function of peripheral T cells (Fallarino et al., 2010).

On the bases of this well described role of mGlu4 receptor in the immune system, we asked whether the same receptor can also mediate a protective effect in neuroinflammation directly at the CNS. Hence, we have investigated in more detail the role of mGlu4 receptor in CNS cells, focusing specifically on its role in oligodendrocyte survival. The effects of mGlu4 receptor activation on different glial cell types and the impact on oligodendrocyte survival have been analyzed.

## Materials and methods

### Drugs and reagents

Cell culture plastics were provided by BD Falcon (Milan, Italy.) Media, media supplements, serum, trypsin, poly-D-Lysine, buffers and antibiotics were from Invitrogen Srl (Milan, Italy). All components and growth factors for chemically defined medium were from Sigma-Aldrich (St. Louis, MO, USA). L-(+)-2-Amino-4-phosphonobutyric acid (L-AP4), cyclopropyl-4-phosphonophenylglycine (CPPG), (-)-N-Phenyl-7-(hydroxyimino)cyclopropa[b]chromen-1a-carboxamide (PHCCC) and kainic acid were from Tocris Cookson Ltd (North Point, UK).

The following primary antibodies were used: mouse anti-O4 (1:40, Sigma-Aldrich), rabbit anti-mGluR4 (1:100, Millipore, Billerica, MA, USA), mouse anti-glial fibrillary acidic protein (GFAP; 1:300 Cell Signaling Technology, Beverly, MA, USA), chicken anti-myelin basic protein (MBP; 1:500, Aves, Tigard, OR, USA), chicken anti-Integrin α-M (1:40, Aves), mouse anti-α-actin (1:5000, Sigma-Aldrich), rabbit anti platelet-derived growth factor (PDGF) receptor (1:40, Santa Cruz) and mouse anti-transforming growth factorβ1 (TGFβ1; R&D Systems, Minneapolis, MN, USA, 2 μg/ml for Western blot and flow cytometry and 5 μg/ml as a neutralizing agent). The following fluorochrome-conjugated secondary antibodies were used: phycoerithryn anti-mouse (1:400) from Santa Cruz Biotechnology (Santa Cruz, CA, USA), Alexa-Fluor 546 anti-mouse and anti-rabbit (1:500, Invitrogen), FITC anti-chicken (1:500, Aves) and Texas red anti-chicken (1:100, Santa Cruz).

### Cell cultures

All animal experimental procedures were carried out in accordance with the directives of the Italian and EU regulations for care and use of experimental animals (DL116/92) and approved by the Italian Ministry of Health. Preparation of glial cell cultures was carried out as previously described (Spampinato et al., 2012) using 1- to 3-day-old Sprague-Dawley rats (Harlan, Udine, Italy). After removal of meninges and isolation of cortices, cells were dispersed by mechanical and enzymatic dissociation using a solution of trypsin diluted in Hank’s Balanced Salt Solution. Cells were plated onto 75 mm^2^ flasks, and maintained in Dulbecco’s Modified Eagle’s medium (DMEM) supplemented with 10% fetal calf serum (FCS), penicillin/streptomycin (100 U/ml-100 μg/ml) and glutamine (2 mM). Confluent cultures at 10–12 DIV were shaken for 2 h at 37°C at 250 RPM to yield microglial cells that were plated and maintained in DMEM with 10% FBS for 5–6 days. Flasks were then more vigorously (450 RPM) shaken for eight additional hours to separate oligodendrocytes from astrocytes. Immature oligodendrocytes were maintained in DMEM-F12 supplemented with penicillin/streptomycin, albumin from bovine serum (BSA; 1%), glucose (1%), insulin (10 ng/ml), apotransferrin (10 ng/ml), putrescine (100 μM), glutamine (2 mM), selenium (30 nM), progesterone (20 nM), PDGF (10 ng/ml), fibroblast growth factors (FGF; 5 ng/ml), T3 (20 ng/ml), T4 (20 ng/ml) for 2 days. PDGF/FGF were removed on the day of treatment. Adherent astrocytes were collected by trypsin digestion, plated in DMEM supplemented with 10% FCS, and used for experiments 6–8 days after re-plating.

### Immunostaining

Cells were fixed in 4% paraformaldehyde, permeabilized when necessary with 0.1% Triton X-100 and saturated with 3% BSA. Cells were then incubated with selective primary antibodies then exposed to fluorochrome-conjugated secondary antibodies. Quantitative analysis of oligodendrocyte morphology was carried out on skeletonized images using a plugin for the freely available image-processing program ImageJ, as described (Arganda-Carreras et al., 2010). This method, that is based on sequential erosion of images, provides topological as well as metric information, including number of branches and mean length of branches.

### Western blot

Cells were harvested in radioimmunoprecipitation assay (RIPA) lysis buffer supplemented with anti-protease and anti-phosphatase cocktail mixes and protein concentration was determined with the Bradford reagent (all from Sigma-Aldrich). Fifty to eighty micrograms of each sample were separated by SDS-PAGE and transferred to nitrocellulose membranes (Hybond ECL, Amersham, Milan, Italy). Membranes were blocked with 3% non-fat milk for 30 min and processed for immunodetection using specific horseradish peroxidase (HRP)-conjugated secondary antibodies (Santa Cruz) and the Immobilon Western Chemiluminescent HRP substrate detection system (Millipore). Densitometric analysis of band intensity was carried out with the Image J Software developed by NIH and freely available on the web.

### PCR and QRT-PCR

Total RNA was extracted from cortical astrocytes with RNeasy Plus Mini Kit (Qiagen, Milan, Italy). Two microgram of total RNA were used for cDNA synthesis, using the Superscript Vilo Kit (Invitrogen) according to manufacturer’s instructions. Two microliter of cDNA were used in each subsequent PCR amplification, in an automatic thermal cycler (MJ Research), using PCR master mix (Invitrogen) and corresponding oligos. PCR amplification was conducted at 95°C for 5 min followed by 30 cycles at 94°C and 55°C for 45 s each and 72°C for 1 min. The final extension step was 10 min at 72°C. PCR amplicons were visualized electrophoretically on a 2% agarose gel in Tris-acetate/EDTA buffer after SYBR Green gel staining (Invitrogen). A single band was always detected. Lack of contaminating genomic DNA was verified using no-RT controls.

Quantitative real time PCR (QRT-PCR) was performed with Rotor Gene Q using QuantiNova Sybr Green PCR Kit (Qiagen) according to manufacturer’s instructions. The melting curves obtained after each PCR amplification reaction confirmed the specificity of the SYBR Green assays.

The following primers were used: GAPDH (F 5′-GCCGCCTGGTCACCAGGGCTG-3′; R 5′-ATGGACTGTGGTCATGAGCCC-3′); QuantiTect Primer Assay (Qiagen) for group III mGluRs (mGluR4, cat. no. QT01081556; mGluR6, cat. no. QT00187880; mGluR7, cat. no. QT00180502; mGluR8, cat. no. QT01079960); S18 (F 5′- GAGGATGAGGTGGAACGTGT-3′; R 5′- GGACCTGGCTGTATTTTCCA-3′). The mGluR4/S18 ratio was estimated using the DDCt method.

### RNA silencing

Cultured astrocytes were transfected with lipofectamine 2000 (Invitrogen) and siRNA for mGluR4 (2.5 nM; FlexiTube, cat. no. SI00266546, Qiagen). As control, parallel cultures were transfected with siRNA Negative Control (cat. no. SI03650318, Qiagen). Cells were exposed to lipofectamine and siRNA in Optimem medium for 4 h at 37°C, and let to recover in DMEM with 5% FBS for 24 h.

### Flow cytometry

Astrocytes and oligodendrocytes were gently harvested with trypsin and fixed with paraformaldehyde 4%. Cells were processed for immunostaining overnight at 4°C with primary antibodies, then exposed to fluorochrome-conjugated secondary antibodies for 1 h at room temperature. Analyses were carried out on a Beckman Coulter flow cytometer.

### Statistical analysis

Data shown are always mean ± SEM of 3–6 independent experiments each run in triplicates or quadruplicates. When reported in the text, *n* refers always to independent experiments. Data were analyzed by one-way Anova followed by Newman-Keuls test for significance. *P* < 0.05 was taken as the criterion for statistical significance.

## Results

### mGlu4 receptor mediates oligodendrocyte differentiation

**Figure 1:**
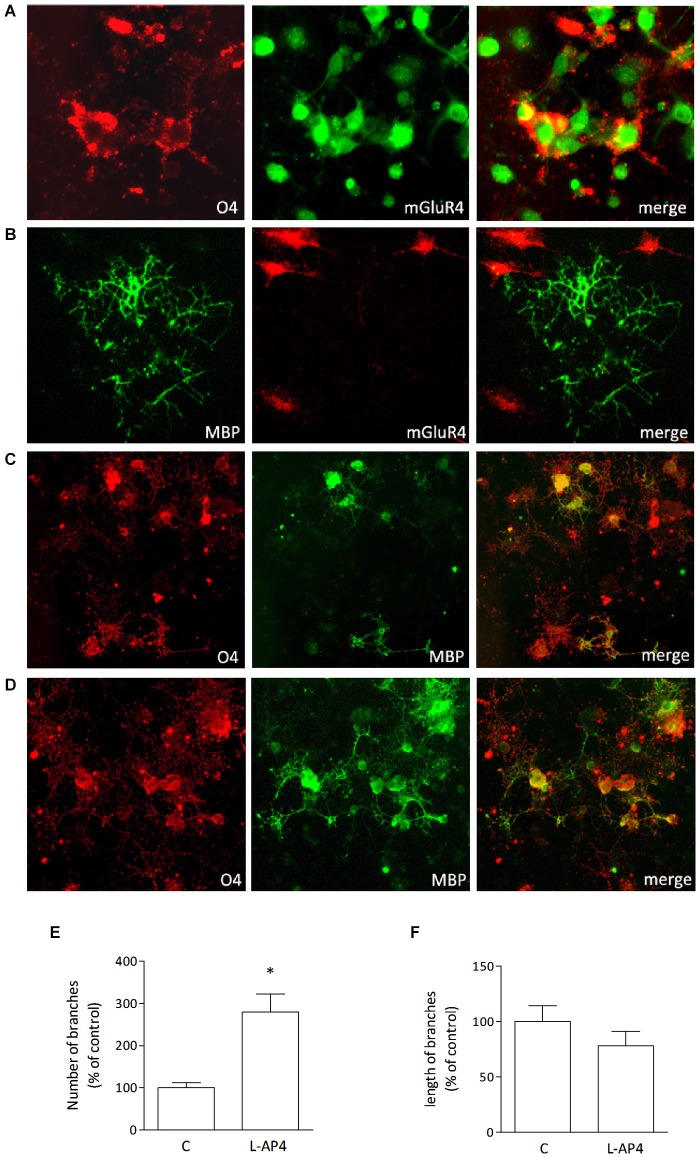
**Oligodendrocytes express mGlu4 receptor and respond to L-AP4 with enhanced differentiation**. Oligodendrocytes that stain positively for O4 (red; **A**) express also mGlu4 receptors (green; **A**). Co-labeling of mGlu4 receptor and MBP (**B**, right panel) reveals that more mature oligodendrocytes (MBP immunopositive **(B)**, left panel) do not express mGlu4 receptor (**B**, middle panel). Treatment with the mGlu4 receptor agonist, L-AP4 (50 μM, for 48 h), accelerates oligodendrocyte differentiation as revealed by their morphology **(D)**, with enhanced initial branching and increased labeling with MBP, as compared to control conditions **(C)**. Scale bar = 10 μm. Quantification of branching is reported and expressed as number of branches **(E)** and length of branches **(F)**. Both values are expressed as % of control and each bar represents mean ± SE of six different fields analyzed in three samples from two independent experiments. *p < 0.05 vs. control by Student’s *t*-test.

Immature oligodendrocytes were maintained in culture in chemically defined medium supplemented with PDGF and FGF and grown for 2–3 days. These cultures, O4-positive, express mGlu4 receptor as detected by immunocytochemical analysis (Figure 1A). However, more mature oligodendrocytes, that are maintained in differentiating medium for 6–7 days prior to PDGF/FGF deprivation and that stain positively for MBP (Figure 1B) do not express mGlu4 receptor.

Treatment of immature cultures with the group III mGlu receptor agonist L-AP4, (50 μM, for 48 h) accelerates oligodendrocyte differentiation as revealed by their morphology, with enhanced branching and increased labeling with anti-MBP (Figure 1D). Vehicle-treated cultures are shown in Figure 1C for comparison. Quantitative analysis reveals that L-AP4 treatment increases branch number (280 ± 42 vs. 100 ± 12%; Figure 1E) whereas the mean length of branches is not modified (100 ± 14 and 78 ± 13% for control and AP4-treated, respectively; Figure 1F). Data are expressed as % change from control values and are from two independent experiments in which six fields in three samples were analyzed.

### Astrocytes play a main role in mGlu4 receptor-mediated oligodendrocyte survival

**Figure 2:**
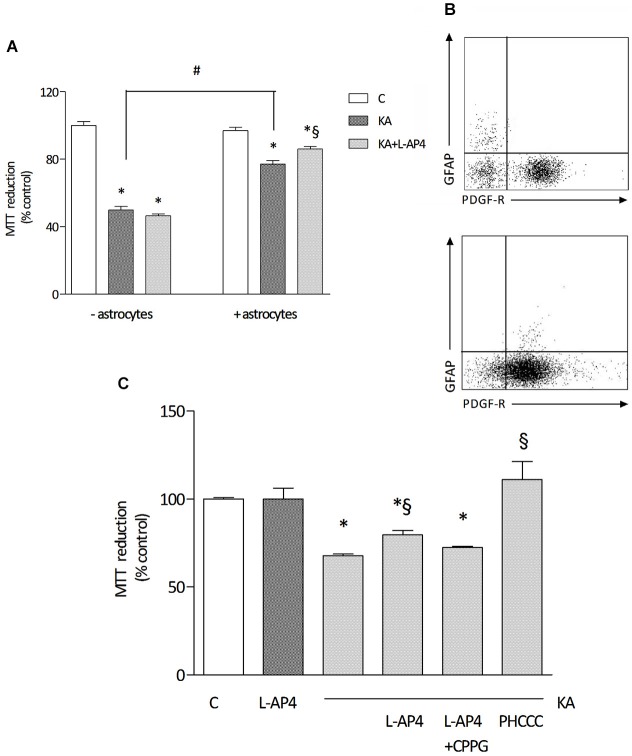
**The effect of L-AP4 on kainic acid-induced oligodendrocyte death depends on astrocytes**. Panel **(A)** reports the different response of a 24 h treatment with 1 mM kainic acid (KA) in pure oligodendrocyte cultures (− astrocytes) or in oligodendrocyte cultures containing a low percentage of astrocytes (+ astrocytes). Group III mGlu receptor agonist, L-AP4 (50 μM), was added, 30 min before treatment with kainic acid **(A)**. Oligodendrocyte viability was measured by the MTT proliferation assay. In **(B)**, representative plots of the characterization of cell population showing 1.8% (upper panel) and 7.9% (lower panel) GFAP immunopositive cells and more than 90 and 80%, respectively, PDGF-R expressing cells, as by flow cytometric analysis. In **(C)**, the effect of a 15 min pre-treatment with the selective mGlu4 receptor antagonist CPPG (100 μM) on L-AP4 reduction of kainic acid-induced oligodendrocyte death. The last bar on the right shows the effect of a 24 h treatment with the positive allosteric modulator of mGlu4 receptor, PHCCC (30 μM for 24 h), on kainate toxicity. Data in **(A)** and **(C)** are mean ± SE of three to four independent experiments, with four replicates per treatment group. *p < 0.05 vs. control, #*p* < 0.05 between indicated groups and §*p* < 0.05 vs. kainic acid by one way ANOVA, followed by Newman-Keuls multiple comparison test.

For toxicity studies, oligodendrocytes at early stages of differentiation, and thus positively staining for both O4 and PDGF receptor, were used. Exposure to 1 mM kainic acid for 24 h induces a marked reduction of cell viability, detected by MTT assay, an effect that is significantly more pronounced in pure oligodendrocyte cultures (49.8 ± 2.2% of control) compared to cultures with a low level of astrocyte contamination (77.2 ± 2.0% of control; *n* = 3–4 independent experiments; Figure 2A). Characterization of the cell population by flow cytometry, revealing respectively <2% (Figure 2B, upper panel) and about 10% (Figure 2B, lower panel) of GFAP-positive cells in the oligodendrocyte culture is reported.

Pretreatment with L-AP4 (50 μM; added 30 min before kainic acid) reduces kainate-induced toxicity. Interestingly, this effect is observed only when oligodendrocytes are grown in the presence of astrocytes (as described above, 86.0 ± 1.6% of control), but not in oligodendrocyte cultures that do not allow astrocyte contamination and that are more sensitive to kainate damage (46.5 ± 1.2% of control; Figure 2A).

The protective effect of L-AP4 against kainate-induced oligodendrocyte death is abrogated by the specific mGlu4 receptor antagonist CPPG (100 μM; 72.4 ± 0.6% vs. 79.5 ± 2.7% of control with L-AP4 and kainate; *n* = 3; Figure 2C) and mimicked by the positive allosteric modulator of mGlu4 receptor, N-Phenyl-7-(hydroxyimino)cyclopropa[b]chromen-1acarboxamide (PHCCC; 30 μM; 111 ± 1.6% of control; Figure 2C) that, *per se*, non significantly, increases by 15% cell viability (not shown).

**Figure 3:**
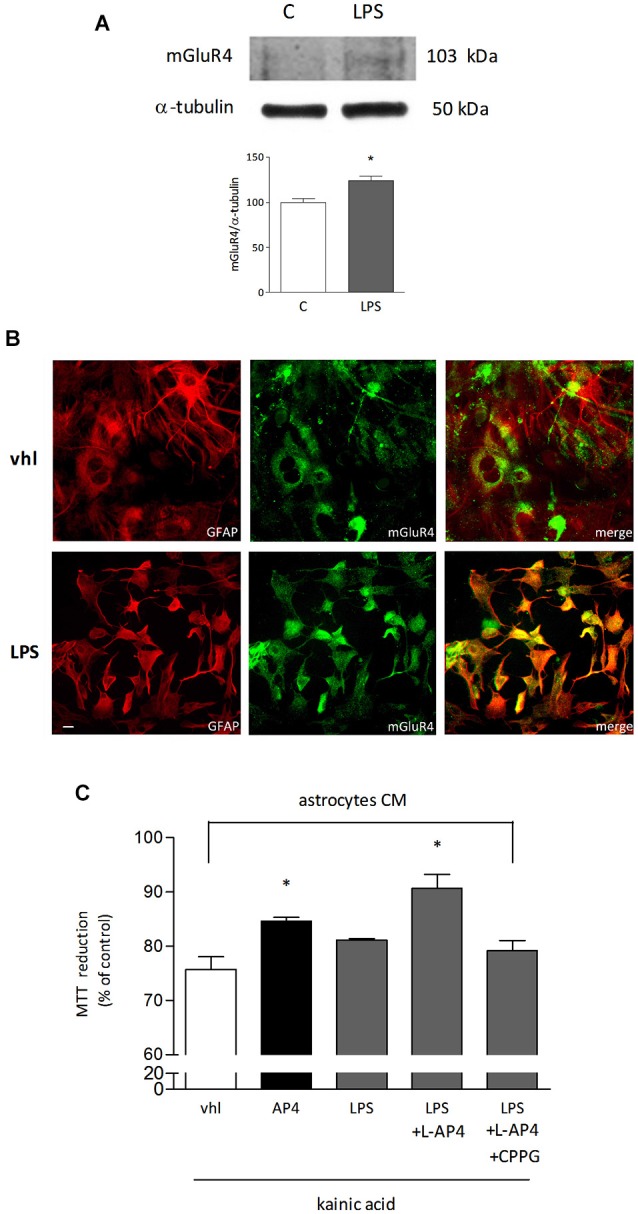
**mGlu4 receptor is expressed in astrocytes and mediates enhanced oligodendrocyte viability**. mGlu4 receptor expression in cultured astrocytes is enhanced by exposure for 48 h to LPS (1 μg/ml), as revealed by western blot analysis **(A)** and immunocytochemistry **(B)**. Representative bands of mGlu4 and α-tubulin are reported and bars showing densitometry result from three independent determinations **(A)**. Co-immunostaining for mGlu4 receptor (green) and GFAP (red) is shown **(B)**. In **(C)**, assessment of the viability of oligodendrocytes challenged with kainic acid (1 mM for 24 h) after transferring of conditioned medium (CM) obtained from astrocytes, under basal or LPS-stimulated conditions, exposed for 2 h, prior to washing and medium collection for the following 18 h, to L-AP4 (50 μM) and the mGlu4 receptor antagonist CPPG (100 μM). Data are mean ± SE of three independent experiments, each run in quadruplicates. *p < 0.05 vs. all other conditions by Student’s *t*-test **(A)** and one-way ANOVA followed by Newman-Keuls test **(C)**. Scale bar = 20 μm.

**Figure 4:**
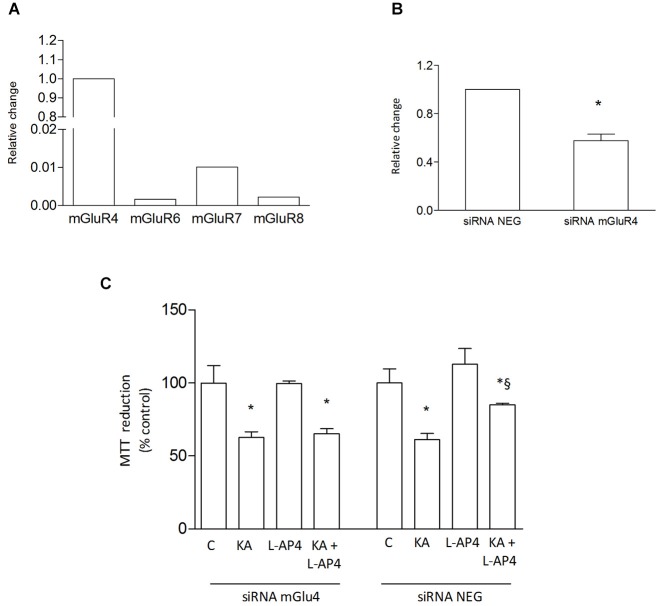
**L-AP4 acts on astrocytic mGlu4 receptor to protect oligodendrocyte against kainate toxicity**. Expression of group III mGlu receptors, mGlu4, mGlu6, mGlu7 and mGlu8 receptors, was analyzed in astrocytes by QRT-PCR **(A)**. Expression of mGlu4 receptor after transfection of cultured astrocytes with mGlu4 receptor siRNA (siRNA mGluR4) is shown and compared to expression in astrocytes exposed to siRNA negative control (siRNA Neg; **(B)**). In **(C)**, the effect of CM from astrocytes treated with L-AP4 following silencing of mGlu4 receptor (siRNA mGlu4) or its negative control (siRNA Neg) on kainate-induced oligodendrocyte death. In **(A)**, data are representative of several independent assays each run in triplicates. Data in **(B)** and **(C)** are mean ± SE from four independent determinations. * *p* < 0.05 vs. respective control; § < 0.05 vs. kainate alone. A *t*-test and one-way ANOVA plus Newman-Keuls test were applied to detect statistically significant differences in **(B)** and **(C)**, respectively.

Since the presence of astrocytes appears necessary for detection of mGlu4 receptor-mediated oligodendrocyte survival, attention has been addressed to this glial cell type. Indeed, mGlu4 receptor is present in astrocytes cultured under basal conditions and its expression is significantly increased in astrocytes activated by exposure to LPS (1 μg/ml for 48 h), as shown by western blot analysis (124 ± 5% of control; *n* = 3; Figure 3A). Similarly, immunocytochemical analysis shows several cells with intense mGlu4 receptor immunolabeling upon LPS treatment (Figure 3B). Transfer of conditioned medium (CM) from astrocytes, exposed for 2 h to L-AP4 (50 μM), before washing and medium collection for the following 18 h, reduces kainate-induced oligodendrocyte toxicity. This effect is already present under basal conditions, but is more pronounced after transfer of CM from LPS-activated astrocytes (84.6 ± 0.7 and 90.7 ± 2.6% of control, respectively, *n* = 3; Figure 3C). The experimental paradigm in this case is the same described above, but astrocytes are activated by exposure to LPS for a 48 h period that includes the 2 + 18 h of L-AP4 treatment and CM collection, respectively. The effect of L-AP4 is prevented by pre-treatment of astrocytes with the mGlu4 receptor antagonist, CPPG (79.2 ± 1.9%; Figure 3C), that, *per se*, is devoid of any effect. Since L-AP4 is known to activate all class III mGlu receptors and to further support the involvement of mGlu4 subtype, the expression of mGlu4, mGlu6, mGlu7 and mGlu8 receptors in astrocytes has been assessed by QRT-PCR. As shown in Figure 4A, astrocytes, in our culture conditions, express mGlu4 receptor and only very low levels of mGlu7 receptor, while mGlu6 and mGlu8 receptors are undetectable (representative data from one experiment run in triplicates are shown). Data were confirmed by gel electrophoresis following RT-PCR (not shown). Furthermore, silencing of mGlu4 receptor, that yields about a 40% reduction of its expression, as detected by QRT-PCR (Figure 4B), dramatically affects the ability of CM from L-AP4-treated astrocytes to protect oligodendrocytes against kainate toxicity (65.2 ± 3.6 and 62.6 ± 3.9% vs. 84.9 ± 1.1 and 61.2 ± 4.2% for kainate and L-AP4 vs. kainate alone, under silencing and non-silencing conditions, respectively; *n* = 4; Figure 4C).

### Transforming growth factor β1 (TGF-β) is a mediator of mGlu4 receptor-induced oligodendrocyte survival

**Figure 5:**
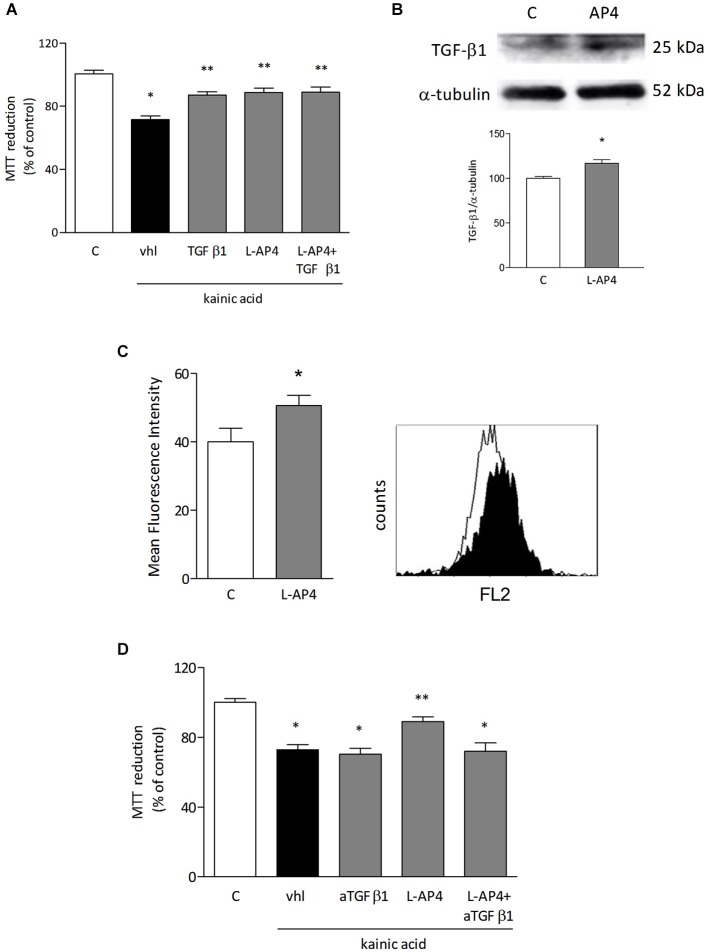
**TGF-β1 is involved in the protective effect of L-AP4 against kainate-induced oligodendrocyte death**. Oligodendrocytes were treated with TGF-β1 (10 ng/ml), L-AP4 (50 μM) or both, 30 min prior to exposure to kainic acid (1 mM for 24 h). Cell viability was assessed by the MTT assay **(A)**. TGF-β1 expression in astrocytes assessed by western blot analysis under basal and L-AP4-stimulated conditions **(B)**. The intracellular content of TGF-β1, assessed by flow cytometry in astrocytes exposed to LPS (0.1 μg/ml for 24 h) and treated with 50 μM L-AP4 for 6 h in the presence of brefeldin **(C)**. Mean fluorescence intensity of all positive events is reported. A representative plot showing basal (empty histogram) and L-AP4-treated (filled histogram), TGF-β1 positive cells is also shown **(C)**. In **(D)**, oligodendrocyte viability after exposure to kainic acid (1 mM for 24 h) assessed in the presence of anti-TGF-β1 antibody (5 μg/ml), after transferring of CM obtained from astrocytes activated with LPS (1 μg/ml for 24 h) and treated with L-AP4 (50 μM) for 2 h prior to medium collection for 18 h. Data are mean ± SE of 4–6 independent experiments, each with three to four replicates. *p < 0.05 vs. respective control and **p < 0.05 vs. kainic acid (vhl) in a and vs. all other kainic acid-treated conditions (vhl; aTGF-β1; L-AP4 + aTGF-β1) in **(D)**. Significance was assessed by ANOVA and Newman-Keuls test. In **(B)** and **(C)** a Student’s *t-test* was applied.

In order to identify soluble factors released by astrocytes responsible for the observed protective effect, attention was focused on TGF-β1. Treatment with TGF-β1 (10 ng/ml) for 24 h protects in fact oligodendrocytes against kainate-induced cell death and simultaneous treatment of oligodendrocytes with 50 μM L-AP4 and 10 ng/ml TGF-β1 produces a non additive effect (71.4 ± 2.5% for kainate alone vs. 88.8 ± 2.7, 87.1 ± 2.0, 88.8 ± 3.4% for kainate plus L-AP4, TGFβ1, and L-AP4 and TGFβ1, respectively; *n* = 6; Figure 5A). Exposure of astrocytes to 50 μM L-AP4 for 24 h increases the expression of TGF-β1 as shown by western blot analysis (Figure 5B). Similarly, flow cytometry reveals that the intracellular content of TGF-β1, measured in the presence of brefeldin (1 μg/ml), is increased upon exposure of LPS-pretreated astrocytes to L-AP4 for 6 h (50.6 ± 3.0 vs. 40.0 ± 4.0; *n* = 3; Figure 5C). The 6 h time point was chosen in order to detect initial accumulation of TGFβ1 following AP4 stimulation, trying to avoid too long exposure to brefeldin that may be toxic to astrocytes. To ascertain whether an enhanced release of TGF-β1 from astrocytes could account for the protective effect of L-AP4, kainate toxicity was analyzed in oligodendrocytes exposed to L-AP4-treated astrocyte CM, in the presence of a neutralizing anti-TGF-β1 antibody (5 μg/ml), during kainate exposure. Under these conditions, a significant reduction of L-AP4 protective effect is observed (72.0 ± 4.8 vs. 89.0 ± 2.8% of control; *n* = 4; Figure 5D).

### Microglia is not involved in the protective effect of L-AP4 on kainate-induced oligodendrocyte death

**Figure 6:**
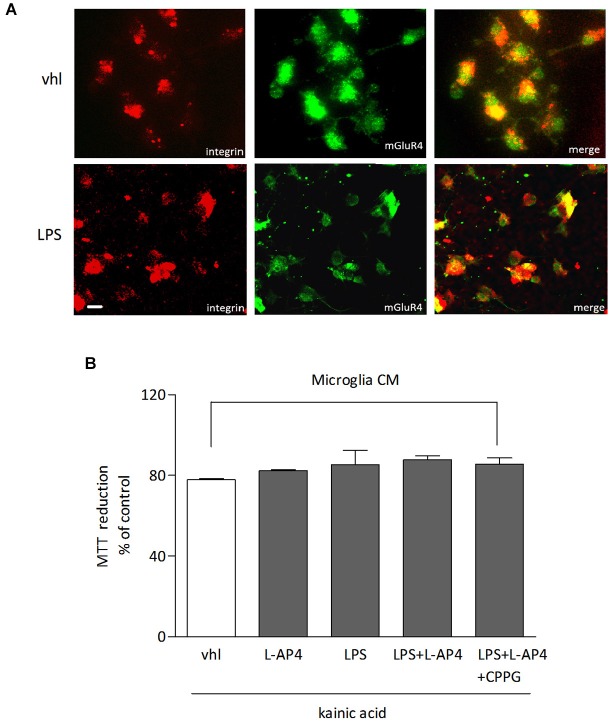
**Lack of effect of L-AP4-treated microglia CM on oligodendrocyte survival**. mGlu4 receptor expression in microglia under resting conditions (vhl) or upon exposure to LPS (0.1 μg/ml) for 48 h as revealed by immunocytochemistry **(A)**. Microglia cells were labeled with an antibody against the specific microglia marker integrin. In **(B)**, oligodendrocyte survival after 24 h exposure to kainic acid (1 mM for 24 h) is not modified by transferring of CM from microglia, under resting or LPS-activated conditions, treated or not with L-AP4, 50 μM for 2 h, prior to medium collection for additional 18 h. Bars represent mean ± SE from two separate experiments. Scale bar = 10 μm.

The role of microglia as a potential target of L-AP4 effect was analyzed in microglial cultures, under resting and LPS-activated conditions. mGlu4 receptor is expressed in microglia independently of the state of activation, as shown by co-immunostaining with anti-mGlu4 receptor and anti-integrin, a marker of microglia (Figure 6A). However, transfer of L-AP4-treated CM from resting and activated microglia does not modify oligodendrocyte viability (*n* = 3; Figure 6B).

## Discussion

A role for mGlu receptors in demyelinating diseases has long been suggested and has found support mainly in descriptive studies reporting enhanced expression of mGlu receptors in MS lesions (Geurts et al., 2003, 2005; Newcombe et al., 2008), but also in *in vivo* studies showing a protective effect of drugs acting on mGlu receptors in EAE animals (Besong et al., 2002; Fazio et al., 2008; Fallarino et al., 2010). In this regard, particularly interesting is the involvement of mGlu4 receptor, whose stimulation by L-AP4 results in improved recovery rate from EAE, whereas treatment with the mGlu4 receptor positive allosteric modulator (PAM), perhydrocyclopentanophenanthrene (PHCPP), reduces the frequency and severity of relapses in EAE mice (Fallarino et al., 2010). More recently, cinnabarinic acid, that acts as an orthosteric agonist of mGlu4 receptor (Fazio et al., 2012), was shown to exert protective activity against EAE by modulating immune function (Fazio et al., 2014). In addition, EAE appears more severe in mice lacking mGlu4 receptor, further supporting a main role for this receptor subtype in the demyelinating process. Of note, the work by Fallarino et al. (2010) demonstrated that mGlu4 receptor expressed in peripheral dendritic cells mediates immunoregulatory effects, affecting T cell development and adaptive immunity. In the present study we have extended the analysis of the role of mGlu4 receptors to glial cells and show that this receptor is expressed in astrocytes, oligodendrocytes and microglia. However, activation of astrocytic mGlu4 receptor appears primarily involved in enhanced survival of oligodendrocytes exposed to an excitotoxic challenge. Hence the ability of mGlu4 receptor ligands to affect neuroinflammation acting on immune system cells, is complemented with the present data showing an astrocyte-mediated effect on oligodendrocyte survival. In our opinion, this observation deserves great attention as mGlu4 receptor is identified as a novel target able to mediate various effects, at different sites, to protect from demyelination. Interestingly, in fact, other pharmacological approaches, such as antagonists of ionotropic glutamate receptors, have shown increased oligodendrocyte survival, but no effect on neuroinflammation (Pitt et al., 2000).

Expression of mGlu4 receptor in oligodendrocytes has already been demonstrated and appears developmentally regulated (Deng et al., 2004), consistent with a role for this receptor in oligodendrocyte differentiation. Accordingly, activation of mGlu4 receptor by L-AP4 results in earliest appearance of a differentiated phenotype, with enhanced branching and MBP staining, an effect that is evident up to 48 h after treatment, but not thereafter, when the level of differentiation is comparable in control and L-AP4-treated cultures. Interestingly, a detailed analysis of branch formation reveals increased sprouting by mGlu4 receptor stimulation correlates with increased number of branches, but the ability of oligodendrocytes to increase their branch length is not modified.

Despite its expression in oligodendrocytes, astrocytic mGlu4 receptor appears necessary to protect oligodendrocytes against the excitotoxic insult. The strict interplay between astrocytes and oligodendrocytes is supported by the observation that kainate-induced toxicity is attenuated by stimulation of mGlu4 receptor only in a mixed culture containing oligodendrocytes and astrocytes, but no effect is observed when L-AP4 acts directly on oligodendrocytes. Such a protective effect is achieved also when transferring CM from L-AP4-stimulated astrocytes, under basal conditions, but more effectively, after activation with LPS. Of note, under these conditions, mGlu4 receptor expression is slightly upregulated, suggesting a function for this receptor in those circumstances that produce astrocyte reactivity. Activation of mGlu receptors, including mGlu4, in astrocytes, is reported to cause neuroprotection (Yao et al., 2005; Zhou et al., 2006; Corti et al., 2007) and here we show that astrocytes mediate also mGlu4 receptor-enhanced survival of oligodendrocytes. This is consistent with the greater toxicity caused by kainate in pure oligodendrocyte cultures, whereas the presence of astrocytes is *per se* protective.

It is then plausible that soluble factors released by astrocytes mediate L-AP4-increased oligodendrocyte viability. In this regard, our attention has focused specifically on TGFβ1, due to the main role played by this growth factor in astrocyte-mediated neuroprotection (Bruno et al., 1998; Brionne et al., 2003; Vivien and Ali, 2006; Caraci et al., 2012). We show here that TGFβ1 is increased upon L-AP4 activation, protects oligodendrocytes from kainate-induced toxicity and this effect is attenuated in the presence of a neutralizing anti-TGFβ1 antibody. Consistent with this observation, TGFβ1 is increased by cinnabarinic acid treatment in brain infiltrating leukocytes of wild type, but not, mGlu4 receptor knockout EAE animals (Fazio et al., 2014). The role of TGFβ1 in neuroinflammation is however rather controversial. In fact, the reported amelioration of clinical symptoms of EAE following TGFβ1 treatment (Johns et al., 1991; Kuruvilla et al., 1991), the enhanced disease severity following administration of anti-TGFβ1 neutralizing antibodies (Johns and Sriram, 1993), and the protection against EAE induction (Cautain et al., 2001; Blazevski et al., 2013) are counterbalanced by the observation that TGFβ1 is overexpressed in the early phases of EAE, long before the appearance of clinical signs, and may create a permissive environment for T cell infiltration and the onset of autoimmune inflammation (Luo et al., 2007). Our data demonstrate that TGFβ1 production induced by mGlu4 receptor stimulation protects oligodendrocyte against an excitotoxic insult, further reinforcing the concept that TGFβ1 effects can be site-specific and are protective when directed to CNS cells. To our knowledge, no evidence correlates TGFβ1 to oligodendrocyte viability, with the exception of the reported TGFβ1-induced apoptosis in murine OLI-neu cell line (Schuster et al., 2002; Schulz et al., 2008), a cellular model very different from the primary oligodendrocytes used in the present study.

Finally, mGlu4 receptors are expressed in microglia and their stimulation does not modify the viability of oligodendrocytes undergoing excitotoxicity, at least in our experimental conditions. It has been previously reported in fact that L-AP4 reduces microglial neurotoxicity (Pinteaux-Jones et al., 2008) and prevents inhibition of microglial-induced oligodendrocyte precursor cell proliferation (Taylor et al., 2010). Interestingly, however, L-AP4 was shown to reduce the inflammatory response (Taylor et al., 2003), and to decrease MHC-class II immunostaining in the spinal cord of MBP immunized rats (Besong et al., 2002). This suggests a protective role of L-AP4 during the inflammatory response, as MHC-class II positive microglia cells are present in EAE and peak at the onset of clinical symptoms (Murphy et al., 2010).

In conclusion, the present data demonstrate that mGlu4 receptors exert different functions in glial cell types, including enhanced oligodendrocyte viability, an effect that appears well in line with the reported neuroprotective effects ascribed to this receptor (reviewed in Williams and Dexter, 2013). Importantly, the effects mediated by mGlu4 receptors expressed on glial cells appear to be complementary to those exerted in the immune system (Fallarino et al., 2010), further confirming that targeting mGlu4 receptors may be effective to contrast neuroinflammatory conditions.

## Conflict of interest statement

The authors declare that the research was conducted in the absence of any commercial or financial relationships that could be construed as a potential conflict of interest.
